# Safety and efficacy of n-3 fatty acid-based parenteral nutrition in patients with obstructive jaundice: a propensity-matched study

**DOI:** 10.1038/s41430-018-0256-1

**Published:** 2018-07-13

**Authors:** Qiong Gong, Peng Zhu, Binhao Zhang, Chang Shu, Zeyang Ding, Jingjing Wu, Bixiang Zhang, Xiao-ping Chen

**Affiliations:** Institute of Hepato-Pancreato-Biliary Surgery center, Tongji Hospital, Tongji Medical College, Huazhong University of Science and Technology, Wuhan, 430030 China

**Keywords:** Malnutrition, Clinical trial design

## Abstract

**Background:**

It is reported that lipid emulsion enriched in n-3 fatty acids (FAs) helps us to improve postoperative recovery for surgical patients with biliary tract disease. Its role for postoperative patients with obstructive jaundice is as yet unclear. The object of this study was to evaluate the safety and efficacy of n-3 fatty acid-based parenteral nutrition (PN) for patients with obstructive jaundice following surgical procedures.

**Methods:**

Data were collected from patients with obstructive jaundice who received PN, including n-3 PUFA-enriched lipid emulsions and standard non-enriched lipid emulsions (e.g., soybean oil). We then calculated a propensity score, the probability of receiving different PN, by the propensity score matched (PSM) method. After matching, we compared isonitrogenous total PN with 20% Structolipid and 10% n-3 fatty acid (Omegaven, Fresenius-Kabi, Germany) (treatment group) to Structolipid alone (control group) for 5 days postoperatively, in the absence of enteral nutrition.

**Results:**

Before the propensity score matching, there were 226 patients enrolled. After propensity score stratification, 108 cases remained, and all covariates were balanced. Among matched patients with PN, patients in the control group were at a higher risk for long-term jaundice recovery (12.9 ± 8.5 VS 16.4 ± 7.9 *P* = 0.029), lower velocity of reduction in jaundice (*P* = 0.045), and lower pre-albumin (*P* = 0.002). No significant difference as found in terms of comorbidities, white blood cell (WBC), albumin and other aspects.

**Conclusion:**

PN with n-3 PUFA-enriched lipid emulsions was safe and effective in accelerating jaundice recovery for patients after surgical procedures. This trial was registered at clinicaltrials.gov as NCT03376945.

## Introduction

Obstructive jaundice is common in liver and biliary disease. Patients with obstructive jaundice are always significantly malnourished [1]. Jaundice is often associated with malnutrition because of changes in intermediary metabolism [2]. Diminished food intake in patients with obstructive jaundice is associated with the degree of hyperbilirubinemia. Biliary drainage can improve food intake and biochemical derangements [3]. Furthermore, nutrition conditions may affect liver function because of lack of essential nutrients for the liver. Hence, it is important for a patient with obstructive jaundice to sustain good nutrition status to avoid postoperative complications.

Surgical procedures in patients with obstructive jaundice can result in increased morbidity and mortality. Several studies have shown that hyperbilirubinemia may lead to poor clinical outcomes [4]. While parenteral nutrition (PN) has been used to prevent malnutrition during the post-surgical phase, it may promote severe hepatic complications and inflammation [5]. Patients with poor nutrition status have greater risk of developing morbidity or mortality after surgery procedures compared with well-nourished patients [6]. This leads to a surgical dilemma: balancing increased mortality and morbidity [7]. A new nutrient supply method for patients with liver disease needs to be addressed urgently to improve hepatic function during the post-surgical management. Lipid emulsion of fatty acid (FA) (n-3 to n-6 = 3:1) was reported to alleviate inflammatory responses in patients with a variety of diseases following major surgery compared with traditional lipid emulsions enriched in n-6 FA [8].

Few studies have concentrated on the relationship between PN enriched in n-3 FAs and comorbidities after surgical procedure as well as recovery. Furthermore, conflicting results have been found in studies of n-3 FAs supplements [9]. One of the studies found that administration of lipid emulsions was not related to improved clinical outcomes in colorectal cancer surgery patients [10]. Another study found that n-3 FAs enriched PN accelerated liver regeneration and functional recovery in mice with hepatic steatosis post-surgery [11]. Lipid composition plays an important role in liver function tests (LFTs) associated with PN, and fish oil intravenous lipid emulsions diminish disturbances of LFT in patients [12, 13]. Because of these discrepancies, we designed a matched cohort trial. The aim of this study was to explore the effect of PN enriched in n-3 FAs given to patients with obstructive jaundice as well study the side effects.

## Methods

### Study design and oversight

This cohort study was a pragmatic, retrospective, single-center, matched, clinical trial from May 2014 to June 2017. The trial included obstructive jaundice patients ≥20 days who received PN treatment for ≥5 days for maintenance and improvement of their nutrition status in the perioperative period. And associated therapy of the patient’s nutrition and energy supply was delineated in Supplemental Table 2. All patients received PN along with the three macronutrients, compounded in an “All-In-One” manner, including amino acids, lipids, glucose, electrolytes, and micronutrients (trace elements and vitamins). The administration was performed with a perfusion pump through a central line within 24 h. All patients were observed for more than 1 week after surgery. Twenty percent Structolipid and 10% n-3 fatty acid (Omegaven, Fresenius-Kabi, Germany) were applied to the n-3 fatty acid treated group, but only Structolipid (n-6 FA) to the control group for five consecutive days postoperatively. Non-protein energy requirement was defined as 25 kcal/kg per day. Glucose made up appropriately 60% of non-protein energy requirements, and the remaining was provided by lipid emulsion. The supply of glucose, lipid, and amino acids was based on 3 g/kg, 0.8 g/kg, and 1.2 g/kg per day, respectively. The concentration of amino acids, enriched in branched-chain amino acid, was 10.8%. The n-6/n-3 FA ratio was 7:1 in the control group and 2:1 in the treatment group, respectively. And except the PN, there is no other nutrition therapy. After a primary screen of the 827 patients with obstructive jaundice, 226 cases were enrolled in the study consecutively. Finally, 108 cases were left for the further research after propensity score matching. This study was funded by the State Key Project on Infection Disease of China and the Chinese Ministry of Public Health for Key Clinical Projects. Data from this study were inputted by two physicians of our department independently, then tested against one another.

We measured complications to assess n-3 FAs safety. We evaluated efficacy with the velocity of the serum TBIL clearance, which was calculated according to the formula: (formal TB -TB of current)/time interval. A faster velocity indicated a more capable drug.

### Patients

Inclusion criteria for screening the cases were as follows: (1) the diagnosis of obstructive jaundice must be clear (TBIL > 51.3 µmol/L plus imaging evidence), and the obstruction must be located in the extrahepatic bile duct; (2) duration of jaundice less than 2 weeks; (3) nutritional support is needed; (4) nutritional support was administered during the perioperative period; and (5) drainage treatment was effective.

Exclusion criteria were as follows: (1) contraindication for surgical procedure, including Child-Pugh Classification C, severe hemorrhagic disorders, gastrointestinal hemorrhage, acute infectious disease, active phase of chronic hepatitis B & C, severe circulatory disease, renal failure pre-operation, and other unknown cause; (2) abandonment of treatment; (3) length of stay (LOS) in hospital <5 days; (4) nutrition support <5 days; (5) conservative treatment; (6) incomplete data; and (7) allergic reactions to PN.

Candidates for this study were classified into two groups according to the lipid administered: the n-3 FA PN group (n-3 FAs group) consisted of patients who received lipid emulsion of an n-6/n-3 fatty acid (FAs) ratio of 3:1 during the perioperative period; the control group consisted of patients receiving only normal FA PN low in n-3 FAs. Administration was based on patient status according to the Nutrition Risk Index (NRI). Scores 83.5–97.5 were considered moderately malnourished, while <83.5 suggested severe malnourishment [10, 14]. NRI was calculated in accordance with the following formula: serum albumin (ALB) (g/L) × 1.519 + (current weight/usual weight) × 0.417 × 100. If the patient met at least one of the two criteria, malnutrition was considered [10]: (1) NRI < 100, (2) any of the following: current weight/ideal weight < 95%; serum pre-albumin < 200 mg/L; or serum Alb < 35.0 g/L.

### Data collection

Measurements needed for the study included demographics (race, sex, age, NRI, MNA, and weight) along with clinical (patient ID, diagnosis, LFT, Child-Pugh classification, perioperative blood tests, infection, sepsis, mortality and Clavien-Dindo classification scores [15]), nutritional test (ALB, pre-albumin, total serum protein, lymphocyte count) and analytical outcomes (glucose, creatinine, triglycerides, CRP, and LFTs: AST, ALT, GGT, and BIL). Unfortunately, lipid metabolism data were incomplete for this study. The primary end point was the safety (mainly complications) and efficacy (duration and jaundice) of the therapy. The secondary end point were the changes of the serum biochemical index (including nutrition status and hepatorenal function) and blood routine test (mainly WBC & GRAN).

Blood samples were collected before the surgical procedure at 7:00 AM without food, and at days 1, 3, 5 after surgery. Patients left the hospital after serum total bilirubin was normal.

According to the method of the drainage for the bile duct, we divided the surgical procedure into three classifications: minimally invasive external drainage, laparotomy external drainage, and bile intestinal anastomosis. Surgical procedures were as follows: percutaneous transhepatic cholangial drainage (PTCD), endoscopic nasobiliary drainage (ENBD), laparoscopic cholecystectomy (LC) + laparoscopic common bile duct exploration (LBDE), laparotomy common bile duct exploration (CBDE), open choledochotomy T-tube drainage (OCTD), cholangioenteric anastomosis surgery, and pancreaticoduodenectomy (PD). Sepsis was diagnosed when patients presented at least two of the following symptoms: (1) body temperature >38 °C or <36 °C, (2) tachycardia with heart rate 90 beats/min, (3) tachypnea with paCO_2_ > 32 mmHg or mechanical ventilation, (4) leukocytes > 12 × 10^9^ cells/L [16]. Complications were staged into five grades based on the Clavien-Dindo classification.

### Ethical considerations

Informed consent was considered unnecessary because it was a respective study. This study complied with the Basic Specification of Electronic Medical Cases, issued by China in 2010. The study gained approval from the Ethics Committee for Clinical Pharmacology of Tongji Medical College for data collection.

### Statistical methods

A propensity score matched (PSM) cohort was designed to limit the effects of confounding factors when estimating treatment effects and side effects, given the non-randomized property of the study and the variety of factors that can influence the outcomes. The sample size of 32 was calculated based on the pre-estimated efficacy of primary outcome, considering an 80% power and an alpha of 0.05 to detect a 80% difference in the treatment group and a 50% difference in the control group. The following calculation was used to calculate the sample size: *n*_1_ = *n*_2_ = (*u*_α/2_ + *u*_β_)^2^[π_1_ × (1 - π_1_) + π_2_ × (1 - π_2_)]/(π_1_ - π_2_)^2^. Because of no loss of follow-up the final sample size was assumed as 32 in each group. Data were inputted from the two groups regarding pre-surgical demographics and outcomes from laboratory tests into the SPSS 22.0 system (Illinois, USA). We then chose a caliper value of 0.05 (tested from 0.1 to 0.01) to give two comparable groups related to the above-mentioned factors.

Continuous data of normal distribution are presented as the mean ± SD using Student’s t-test analysis. For non-normal data, we applied non-parametric tests. Categorical data are presented as proportions and frequency and were subjected to the Chi-squared test. The statistical analysis of the database was performed with SPSS 22.0 (Illinois, USA). Most laboratory results of repeated measurements were subjected to intra-group variance analysis. When *P* < 0.05, the difference between two groups was considered statistically significant. In addition, meaningful outcomes were compared twice according to PSM procedure.

## Results

### Patients

**Fig. 1:**
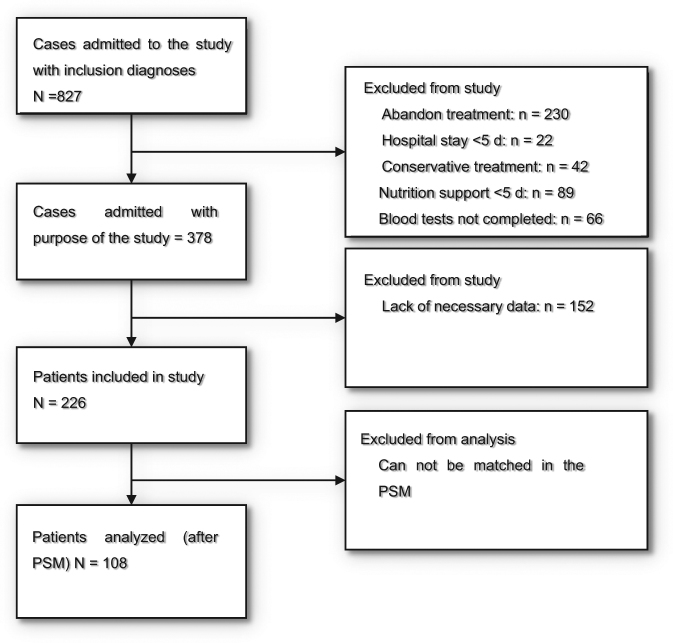
Flow chart of cohort (It shows the process of screening the patients treated or untreated with n-3 FA in the initial and final propensity score matched sample. We screened a total of 827 patients with obstructive jaundice in Tongji Hospital between May 2014 and June 2017. Finally, 108 cases were enrolled into the PSM cohort for the last statistical calculation)

Figure 1 shows the process of screening the patients treated or untreated with n-3 FA in the initial and final PSM. We screened a total of 827 patients with obstructive jaundice in Tongji Hospital between May 2014 and June 2017. Finally, 108 cases were enrolled into the PSM cohort for the last statistical calculation (Fig. 1). The baseline characteristics of the patients before and after matching are summarized in Table 1. Differences between matched and unmatched groups related to surgical procedure as well as the drainage method are displayed in Table 2. Significant imbalances can be detected from the chart before matching and are related to the following factors: BMI, MNA, WBC, ALT, AST, surgical procedure, and drainage methods (*P* < 0.05). This may lead to inaccurate outcomes. All the factors were balanced after the PSM method, resulting in comparable groups of 54 cases (*P* > 0.05).

**Table 1:** Demographic and clinical characteristics

Characteristic pre-operation	Before matched			After matched		
	N-3 fatty group (*n* = 106)	Control group (*n* = 120)	*P*-value	N-3 fatty group (*n* = 54)	Control group (*n* = 54)	*P*-value
Age (years)	58.1 ± 11.2	55.7 ± 12	0.213	56.9 ± 12.4	57.4 ± 11.3	0.846
Male sex No. (%)	36(15.9)	70(31)	0.889	62(58.4)	86(71.7)	0.244
BMI (kg/m^2^)	21.3 ± 2.7	22.1 ± 2.9	0.027	21.7 ± 2.7	21.7 ± 3	0.933
MNA	7.7 ± 1.1	7.5 ± 0.9	<0.001	4.1 ± 0.9	3.9 ± 1	0.253
NRI	90.9 ± 10.8	91.7 ± 10.7	0.694	91.2 ± 7.5	90.4 ± 11.3	0.672
Child-Pugh score(%)			0.154			1
6–7	8(6.6)	6(5.6)		2(3.7)	2(3.7)	
8–10	108(90)	100(94.4)		52(96.3)	52(96.3)	
>10	4(3.4)	0(0)		0(0)	0(0)	
Weight loss (kg)^a^	4.08 ± 3.65	2.76 ± 3.10	0.26	2.8 ± 3	3.1 ± 3.6	0.567
WBC ( × 10^9 / L)	7.97 ± 4.4	6.73 ± 2.87	0.015	7.48 ± 3.91	7.32 ± 3.14	0.821
ALT(U/L)	147 ± 150	104 ± 109	0.017	103 ± 87	114 ± 109	0.549
AST(U/L)	129 ± 129	106 ± 81	<0.001	77 ± 42	91 ± 62	0.176
TP(g/L)	64 ± 8.3	64 ± 5.7	0.46	63.1 ± 7.1	64.3 ± 6	0.352
ALB(g/L)	34.7 ± 6.7	34.2 ± 5.4	0.587	33.8 ± 4.5	33.6 ± 4.3	0.846
PALB (mg/L)	117 ± 56	123 ± 38	0.366	115 ± 48	116 ± 41	0.905
TBIL (µmol/L)	197.2 ± 170.4	192 ± 111.5	0.788	176.1 ± 147.8	179.8 ± 118.8	0.886
DBIL(µmol/L)	158.8 ± 103.1	149.2 ± 123.8	0.524	151.5 ± 109.6	161.3 ± 127.3	0.669
BUN(µmol/L)	4.48 ± 2.23	4.3 ± 2.12	0.535	4.4 ± 2.2	4.1 ± 2.3	0.462
UA(μmol/L)	177.8 ± 72.7	170.4 ± 66.4	0.434	180 ± 62	180 ± 72	0.984

Data are means ± standard deviations or number (%) unless otherwise indicated

^a^Weight loss = usual weight−weight on inclusion

**Table 2:** Comparison of treatments between patients with n-3 FA group and control group

	Before matched			After matched		
treatments	N-3 fatty group (*n* = 106)	Control group (*n* = 120)	*P*-value	N-3 fatty group (*n* = 54)	Control group (*n* = 54)	*P*-value
Surgical procedures			<0.001			0.169
PTCD^a^	28(26.4)	70(58.3)		24(44.4)	22(40.8)	
ENBD^b^	4(3.8)	2(1.6)		2(3.6)	2(3.7)	
LC or LBDE^c^	22(20.7)	10(8.4)		6(11.1)	6(11.1)	
CBDE^d^	12(11.3)	4(3.3)		4(7.4)	5(9.3)	
OCTD^e^	12(11.3)	22(18.3)		13(24)	8(14.8)	
Bypass surgery	2(1.9)	2(1.7)		2(3.6)	2(3.7)	
PD^f^	26(24.5)	10(8.4)		3(5.5)	9(16.7)	
Anastomotic methods			<0.001			0.926
PTCD ± ENBD	32(30.2)	72(60)		26(48.2)	24(44.4)	
External drainage surgery	34(32)	14(11.7)		10(18.5)	11(30.4)	
Bypass surgery	40(37.8)	34(28.3)		18(33.3)	19(35.2)	

*PTCD* percutaneous transhepatic cholangial drainage, *ENBD* bendoscopic nasobiliary drainage, *LC* laparoscopic cholecystectomy, *LBDE* laparoscopic common bile duct exploration, *CBDE* laparotomy common bile duct exploration, *OCTD* open choledochotomy T-tube drainage, *PD* bypass surgery and pancreaticoduodenectomy

In the 108 patients included in the matched group, median age was 57 (45–65) years, median weight was 59 kg (51.7–68.2), and 59.2% of participants were men. All patients suffered from malnutrition, and PN including lipid emulsion was given. No patient died in the matched groups. Infection occurred in 26 patients (16: 10 n-3 FAs: normal), and eight patients suffered sepsis (5:3 FAs:normal).

### Safety

**Fig. 2:**
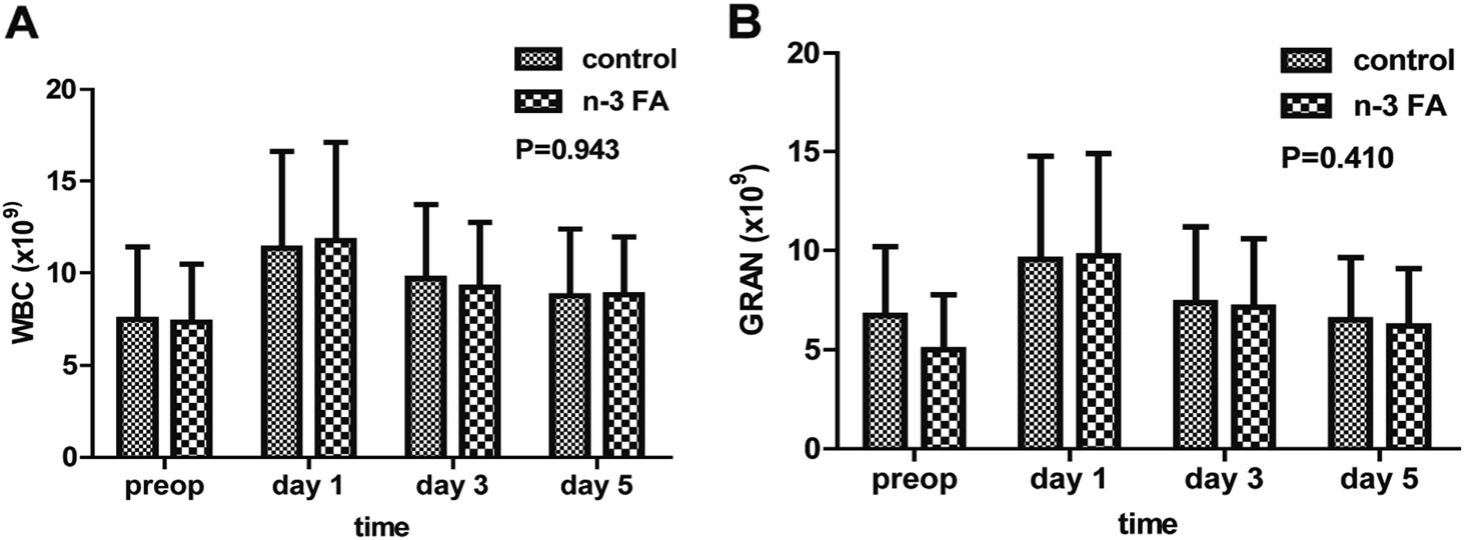
The differences of inflammatory reaction between two groups after operation. There were no differences in WBC or GRAN between two groups (*P* = 0.943, *P* = 0.410), respectively

Among matched 54 patients with jaundice in the n-3 FA group, 21 cases suffered complications, while 23 in 54 patients had complications in the control (*P* = 0.439). There were no significant differences in infectious complications (*P* = 0.177). Likewise, there were no significant differences in sepsis occurrence (*P* = 0.462, supplemental Table 1). There were no differences in WBC or GRAN between two groups (*P* = 0.943, *P* = 0.410, respectively, Fig. 2). Mortality of the two groups was missed, which is included in the grade 5 complications, due to lack of data regarding 4 and 5 grade complications after group matching. Neither group had significant adverse reactions to their nutritional supplementation.

### Efficacy

**Fig. 3:**
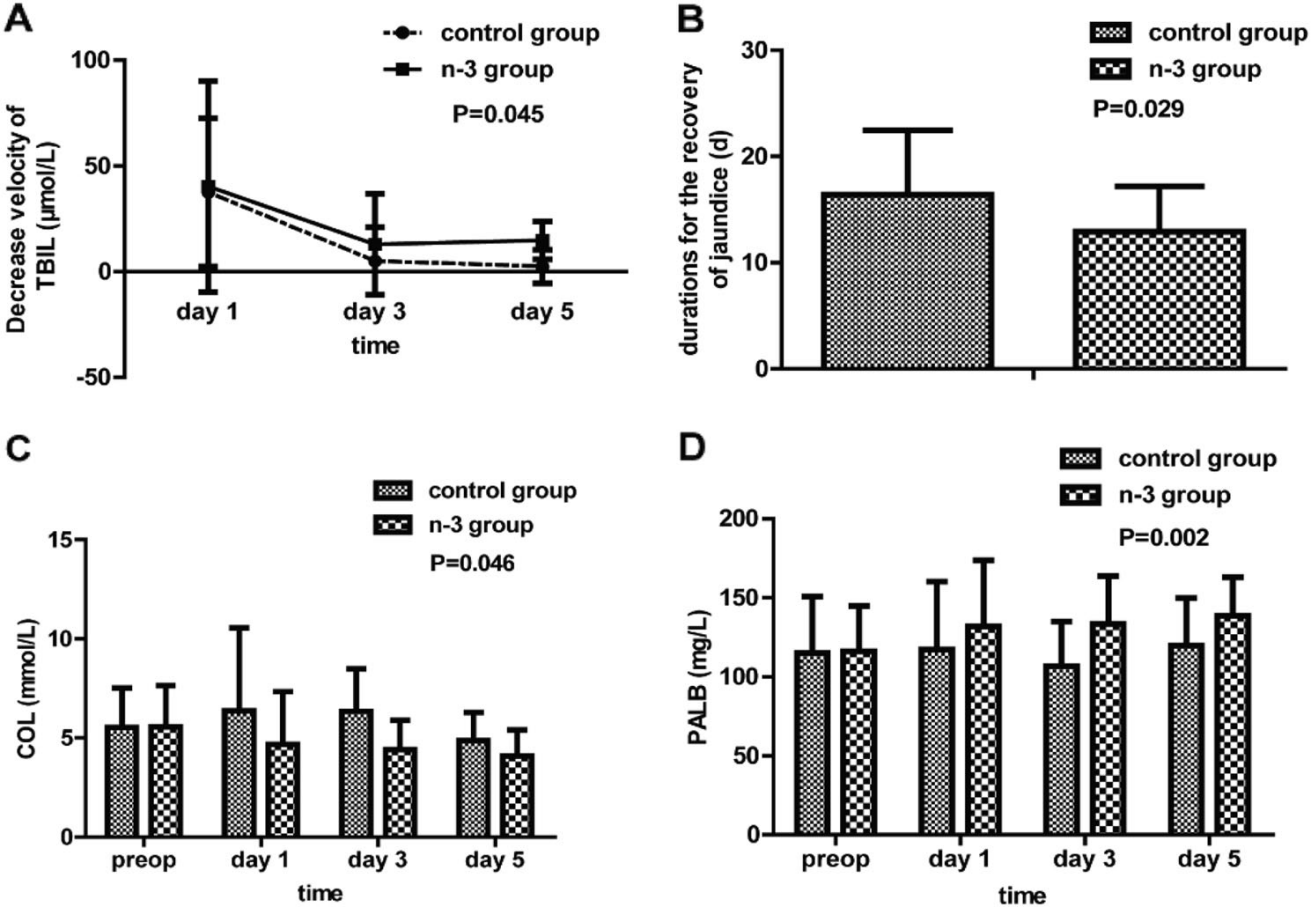
During the postoperative period, patients in the n-3 group had rapid jaundice recovery (**a**) (*P* = 0.045), shorter duration of the jaundice recovery (**b**) (*P* = 0.029), lower cholesterol (**c**) (*P* = 0.045) and higher pre-albumin (**d**) (*P* *=* 0.002),

The velocities of serum total bilirubin clearance on pre-operation day, and the 1st, 3rd, and 5th day after surgery were significantly faster in the trial group than in the control group (*P* = 0.045, Fig. 3); Consistent with this, n-3 FA supplementation led to a reduction in duration of jaundice returning to normal. Patients in the trial group had significant shorter length of cure period after the surgical procedure compared with the control group (12.91 ± 8.46 d vs. 16.39 ± 7.88 d; *P* = 0.029, Fig. 3). In addition, serum cholesterol was significantly higher in the control group [17]. Finally, patients in the treatment group had increased postoperative serum pre-albumin (*P* = 0.005, Fig. 3).

### Secondary outcomes

We found that there was no benefit for renal function in terms of serum creatinine (*P* = 0.010) or GFR (*P* = 0.015) when n-3 FAs were given, although there is no evidence they could impair renal functions (supplemental Fig. 1).

## Discussion

To the best of our knowledge, this is the first study to offer evidence that PN enriched in n-3 fatty acids improved postoperative outcomes for patients with obstructive jaundice following surgical procedures.

The inflammatory process is the unifying principle that is pervasively involved in the pathophysiology of cholestasis and liver disease. Although the evidence is not yet sufficient to make concrete conclusions, there are suggestions that omega‐3 fatty acid supplementation may be helpful in blunting this process. Patients with obstructive jaundice often suffer from malnutrition, absorption disorders, and immunodeficiency. We give these nutrients to patients to help them maintain good nutrition and immunological health, especially when they have been suffering from malnutrition [15, 18, 19].

There is consensus that in many aspects of heart disease, liver disease, and colon cancer, the role of n-3 fatty acid is useful, although effects of n-3 FA have not been related to improved clinical outcomes in some colorectal cancer surgeries. Experts agree that the positive effects of n-3 FA relate to improvements in nutrition and immunity. However, disputes regarding n-3 FA side effects have gone on for a long time, regarding serum lipids, blood uric acid increases, and other clinical outcomes [16]. This is why we focused on this point. We hoped to explore the roles of a lipid emulsion rich in FAs as well as suitable administration time.

According to articles published in recent years, lipid emulsions enriched in n-3 fatty acids (third generation lipids) were developed to modulate the inflammatory response and to provide nutritional support. There is many evidence to suggest that the addition of n-3 FA to normal lipid emulsion would positively modulate inflammation and immunosuppression after surgery [20]. In this paper, the difference between the two groups in terms of jaundice and nutrition status could be detected after 3 days. And the role of the jaundice reduction is more distinct after 5 days administration of n-3 PUFAs. We continue to suggest that PN be given to patients for more than 7 days, since a significant difference has been found in plasma phospholipid profiles after intervention [17].

ESPEN guidelines suggested that optimal PN regimen for critically ill surgical patients should probably include supplemental n-3 fatty acids (Grade C). The evidence-base for such recommendations requires further input from prospective randomized trials. [20, 21]. In a randomized trial, n-3 FA in PN was related to shorter length of hospital stay [22, 23]; n-3 FA have a relatively anti-inflammatory effect when given enterally. In prospective randomized trials, they were associated with decreased pulmonary inflammation, shorter duration of the ventilator treatment, and shorter overall ICU stay [18, 23]. Among healthy subjects, n-3 FAs have been shown to blunt the responses to endotoxin [24]. Taken together, these data suggest that the addition of n-3 fatty acids in PN may improve organ function recovery and reduce hospital stays in patients admitted to the surgical ICU and in patients undergoing major surgery. Although we use the propensity-matched method to avoid the select bias, this method cannot avoid selective bias completely. And this study is not a substitute for randomized control trails and these trends need to be confirmed in adequately powered randomized trials.

The n-3 FAs may function in the following ways: (a) we showed that n-3 FAs could improve nutrition status by increasing pre-albumin (*P* = 0.02); (b) n-3 FAs may reduce the COL after surgery. Due to the improvement of patient’s serum test; (c) n-3 FAs may improve the recovery of patients with jaundice because of the reduction of the duration for the recovery of jaundice after surgery. Mechanistically, n-3 fatty acid enriched in eicosapentaenoic acid and docosahexaenoic acid, was reported to have anti-inflammatory potential through producing thromboxane, 3-series prostaglandins, and 5-series leukotrienes and regulating arachidonic acid pathway. And this leads to a decline of proinflammatory cytokines, including TNF-a, IL-8 and IL-6, and in turn reduces the incidence of postoperative hepatic inflammation. Alternatively, it has been reported to transform membrane fluidity by the regulation of gene transcription such as protein acylation, sterol regulatory element-binding protein (SREBP)-1 and calcium release [6].

The limitations of this paper include the following: this is a single-center report with a limited number of patients. It covered a brief postoperative period for assessing the effects, although the benefit in the long term seen in the n-3 FAs group will not be reversed. In addition, there is no known mechanism to explain n-3 fatty acid-based PN helping to improve postoperative recovery for jaundiced patients, although several mechanisms have been mentioned in this study. Exploring these potential mechanisms should be undertaken in further study, using animal models given n-3 FA-based pN. We provide no evidence for the enhancement of immunity or for the inflammatory factors mentioned in this study. In addition, we do not provide data of regarding interleukin-6, tumor necrosis factor, or c-reactive protein. Finally, enteral nutrition should be studied in a clinical trial. We focused on PN for patients with jaundice following surgery, but attention paid to the role of early enteral nutrition is not enough in this population. Besides, Scr in the n-3 group is significantly higher than that of control group and we may attribute it to the application of n-3 fatty acid. But, Scr concentration in both group belong to normal range (<100μmol/L). That means in general case the drug may not affect the renal function. But patients with poor renal function should be used with caution to avoid abnormal blood creatinine.

In summary, this study provided evidence that n-3 FA-based PN is safe and effective for patients with jaundice following surgery.

## Electronic supplementary material

supplemental fig 1

supplemental table 1

supplemental table 2

supplemental figure and table legend

## References

[1] Gadek JE, DeMichele SJ, Karlstad MD, Pacht ER, Donahoe M, Albertson TE (1999). Effect of enteral feeding with eicosapentaenoic acid, gamma-linolenic acid, and antioxidants in patients with acute respiratory distress syndrome. Enteral Nutrition in ARDS Study Group. Crit Care Med.

[2] Clugston A, Paterson HM, Yuill K, Garden OJ, Parks RW (2006). Nutritional risk index predicts a high-risk population in patients with obstructive jaundice. Clin Nutr.

[3] Gouma DJ, Roughneen PT, Kumar S, Moody FG, Rowlands BJ (1986). Changes in nutritional status associated with obstructive jaundice and biliary drainage in rats. Am J Clin Nutr.

[4] Padillo FJ, Andicoberry B, Naranjo A, Mino G, Pera C, Sitges-Serra A (2001). Anorexia and the effect of internal biliary drainage on food intake in patients with obstructive jaundice. J Am Coll Surg.

[5] Horvatits T, Drolz A, Rutter K, Roedl K, Langouche L, Van den Berghe G (2017). Circulating bile acids predict outcome in critically ill patients. Ann Intensive Care.

[6] Zhang B, Wei G, Li R, Wang Y, Yu J, Wang R (2017). n-3 fatty acid-based parenteral nutrition improves postoperative recovery for cirrhotic patients with liver cancer: a randomized controlled clinical trial. Clin Nutr.

[7] Hughes MJ, McNally S, Wigmore SJ (2014). Enhanced recovery following liver surgery: a systematic review and meta-analysis. HPB: Off J Int Hepato Pancreato Biliary Assoc.

[8] Plusa S, Webster N, Primrose J (1996). Obstructive jaundice causes reduced expression of polymorphonuclear leucocyte adhesion molecules and a depressed response to bacterial wall products in vitro. Gut.

[9] Koller M, Senkal M, Kemen M, Konig W, Zumtobel V, Muhr G (2003). Impact of omega-3 fatty acid enriched TPN on leukotriene synthesis by leukocytes after major surgery. Clin Nutr.

[10] Sorensen LS, Thorlacius-Ussing O, Schmidt EB, Rasmussen HH, Lundbye-Christensen S, Calder PC (2014). Randomized clinical trial of perioperative omega-3 fatty acid supplements in elective colorectal cancer surgery. Br J Surg.

[11] Bozzetti F, Braga M, Gianotti L, Gavazzi C, Mariani L (2001). Postoperative enteral versus parenteral nutrition in malnourished patients with gastrointestinal cancer: a randomised multicentre trial. Lancet (Lond, Engl).

[12] Llop-Talaveron JM, Badia-Tahull MB, Leiva-Badosa E, Ramon-Torrel JM (2017). Parenteral fish oil and liver function tests in hospitalized adult patients receiving parenteral nutrition: a propensity score-matched analysis. Clin Nutr.

[13] Mizuguchi T, Kawamoto M, Meguro M, Hui TT, Hirata K (2014). Preoperative liver function assessments to estimate the prognosis and safety of liver resections. Surg Today.

[14] Juntermanns B, Sotiropoulos GC, Radunz S, Reis H, Heuer M, Baba HA (2013). Comparison of the sixth and the seventh editions of the UICC classification for perihilar cholangiocarcinoma. Ann Surg Oncol.

[15] Dindo D, Demartines N, Clavien PA (2004). Classification of surgical complications: a new proposal with evaluation in a cohort of 6336 patients and results of a survey. Ann Surg.

[16] Licata LA, Nguyen CT, Burga RA, Falanga V, Espat NJ, Ayala A (2013). Biliary obstruction results in PD-1-dependent liver T cell dysfunction and acute inflammation mediated by Th17 cells and neutrophils. J Leukoc Biol.

[17] Popovic T, Ranic M, Bulajic P, Milicevic M, Arsic A, Vucic V (2009). Effects of n-3 fatty acids supplementation on plasma phospholipids fatty acid composition in patients with obstructive jaundice—a pilot study. J Clin Biochem Nutr.

[18] Grossman Y, Barbash IM, Fefer P, Goldenberg I, Berkovitch A, Regev E (2017). Addition of albumin to traditional risk score improved prediction of mortality in individuals undergoing transcatheter aortic valve replacement. J Am Geriatr Soc.

[19] Grimble R (2005). Fatty acid profile of modern lipid emulsions: scientific considerations for creating the ideal composition. Clin Nutr Suppl.

[20] Weimann A, Braga M, Carli F, Higashiguchi T, Hubner M, Klek S (2017). ESPEN guideline: clinical nutrition in surgery. Clin Nutr.

[21] Braga M, Ljungqvist O, Soeters P, Fearon K, Weimann A, Bozzetti F (2009). ESPEN guidelines on parenteral nutrition: surgery. Clin Nutr.

[22] Heller AR, Rossler S, Litz RJ, Stehr SN, Heller SC, Koch R (2006). Omega-3 fatty acids improve the diagnosis-related clinical outcome. Crit Care Med.

[23] Wichmann MW, Thul P, Czarnetzki HD, Morlion BJ, Kemen M, Jauch KW (2007). Evaluation of clinical safety and beneficial effects of a fish oil containing lipid emulsion: data from a prospective, randomized, multicenter trial. Crit Care Med.

[24] Ferguson JF, Mulvey CK, Patel PN, Shah RY, Doveikis J, Zhang W (2014). Omega-3 PUFA supplementation and the response to evoked endotoxemia in healthy volunteers. Mol Nutr Food Res.

