# Supplementary figures and images for: Safety and efficacy of n-3 fatty acid-based parenteral nutrition in patients with obstructive jaundice: a propensity-matched study
Source: Eur J Clin Nutr. 2018 Jul 13;72(8):1159–66. doi: 10.1038/s41430-018-0256-1 (PMC6085574; doi:10.1038/s41430-018-0256-1)

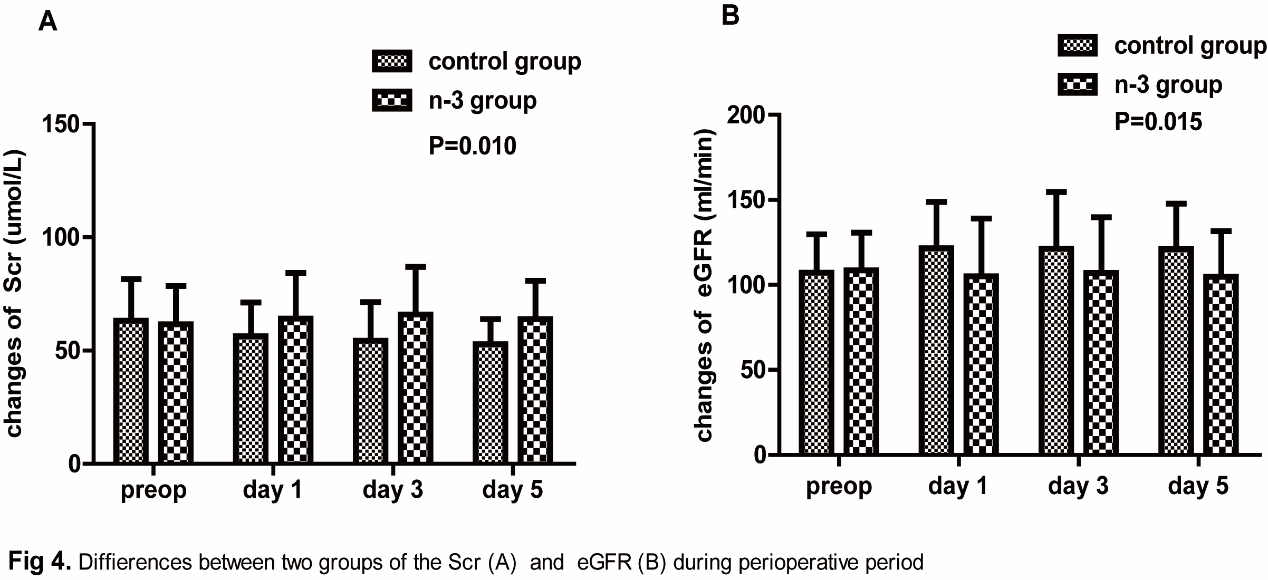


**Supplemental fig 1.**

Supplement: Supplementary file 1 — supplemental fig 1 [file 41430_2018_256_MOESM1_ESM.docx]
