# Supplementary material for: Safety and efficacy of n-3 fatty acid-based parenteral nutrition in patients with obstructive jaundice: a propensity-matched study
Source: Eur J Clin Nutr. 2018 Jul 13;72(8):1159–66. doi: 10.1038/s41430-018-0256-1 (PMC6085574; doi:10.1038/s41430-018-0256-1)
Supplement: Supplementary file 2 — supplemental table 1 [file 41430_2018_256_MOESM2_ESM.docx]

| Supplemental table 1. complications between parenteral nutrition group and control group. | | | |
| --- | --- | --- | --- |
|  | N-3 fatty group (n=54) | Control group (n=54) | P-value |
| Complications (%) |  |  | 0.278 |
| Minor complication ^a^ | 16(69.6) | 10(47.6) | 0.26 |
| Major complication ^b^ | 7(30.4) | 11(52.4) | 0.438 |
| Infectious related complication | 16 | 10 | 0.177 |
| Sepsis | 5 | 3 | 0.462 |
| a. including Clavein-Dindo grade I, II; b. including Clavein-Dindo grade III, IV, V. | | | |
