# Supplementary material for: Safety and efficacy of n-3 fatty acid-based parenteral nutrition in patients with obstructive jaundice: a propensity-matched study
Source: Eur J Clin Nutr. 2018 Jul 13;72(8):1159–66. doi: 10.1038/s41430-018-0256-1 (PMC6085574; doi:10.1038/s41430-018-0256-1)
Supplement: Supplementary file 3 — supplemental table 2 [file 41430_2018_256_MOESM3_ESM.docx]

| supplemental table 2. nutrition and energy support post operation | | | | | | | | |
| --- | --- | --- | --- | --- | --- | --- | --- | --- |
|  | Both Groups | |  | Structural Fat Emulsion |  | Structural + n-3 Fat Emulsion |  | Total Calories (per d) |
| Day | Glucose (Hypertonic glucose injection 20%) | Amino Acids (18-AA 12%) |  | Lipids (Structural Fat Emulsion 10%) |  | Lipids (Structural Fat Emulsion 10%) (omega-3 Fat Emulsion 10%) |  |  |
| 1(/d) | 2.0g/kg | 0.5 g/kg |  | 0.8g/kg |  | 0.54g/kg + 0.26g/kg | | 16kcal/kg |
| 2-5(/d) | 3.0g/kg | 1.2 g/kg |  | 1.0g/kg |  | 0.7g/kg + 0.3g/kg | | 26kcal/kg |
