# Supplementary material for: Safety and efficacy of n-3 fatty acid-based parenteral nutrition in patients with obstructive jaundice: a propensity-matched study
Source: Eur J Clin Nutr. 2018 Jul 13;72(8):1159–66. doi: 10.1038/s41430-018-0256-1 (PMC6085574; doi:10.1038/s41430-018-0256-1)
Supplement: Supplementary file 4 — supplemental figure and table legend [file 41430_2018_256_MOESM4_ESM.docx]

Supplemental table 1. complications between parenteral nutrition group and control group. There were no significant differences in two groups of infectious complications (*P* =0.177). Likewise, there were no significant differences in sepsis occurrence (*P* =0.462), major complication (*P* =0.438) and the minor complication (*P* =0.26).

supplemental table 2. nutrition and energy support post operation. Associated therapy of the patient's nutrition and energy supply follow the table instructions from day 1 to day 5 after surgery.

Supplemental figure 1. Differences between two groups of the Scr (A) and eGFR (B) during perioperative period. There was no benefit for renal function in terms of serum creatinine (P =0.010) or GFR (P =0.015) when n-3 FAs were given. But, they are both in the normal range.
